# Dynamic blood compatibility assessment of coronary stents using an innovative pneumatic blood pump to generate physiological flow rates in small sample chambers

**DOI:** 10.1007/s10856-025-06882-7

**Published:** 2025-04-29

**Authors:** Anne-Sophie Hönicke, Emilia Paz Gross, Simon Beydoun, Felix Pfisterer, Michael Kirschbaum

**Affiliations:** Fraunhofer Institute for Cell Therapy and Immunology, Branch Bioanalytics and Bioprocesses IZI-BB, Potsdam, Germany

## Abstract

According to ISO 10993-4, shear-dependent processes must be considered in the blood compatibility testing of cardiovascular implants, which demands controlled and physiological flow conditions in the test environment. For very small test objects such as coronary stents with a diameter below 5 mm, this is difficult to achieve, as previous test methods are either unable to represent the required flow velocities in small-lumen sample chambers, or can only do so under high pump shear stress, which in extreme cases can mask the effects of the tiny test objects. In this paper, we present a novel concept for dynamic in vitro models based on pneumatically generated blood flow (*AirDrive* technology), which can achieve flow rates and velocities up to 450 ml min^−1^ or 930 mm s^−1^, respectively. This allows for hemocompatibility testing of coronary stents under physiologically high flow rates without generating major shear stress from the pump mechanism. In an initial feasibility study, single coronary stents with different surface finishes (polished/unpolished) were subjected to flow velocities of up to 560 mm s^−1^ in a 3.2 mm diameter sample chamber. Blood samples were collected before and after perfusion, and haematological and coagulation markers were analysed. Unpolished stents elicited higher shear-induced blood responses than polished stents. This demonstrates that our experimental setup is highly sensitive and enables precise and robust investigation of blood compatibility under physiologically relevant flow conditions, even for the smallest objects under investigation. To show the superiority of this novel model in creating high flow rates while maintaining minimal blood damage, the *AirDrive* system was compared to the commonly used roller pump closed-loop system, with the former exhibiting significantly less blood damage. This further confirms that the *AirDrive* technology we present in this paper is of the highest value for developmental or regulatory testing of blood compatibility of small- and medium-sized test objects.

**Graphical Abstract:**

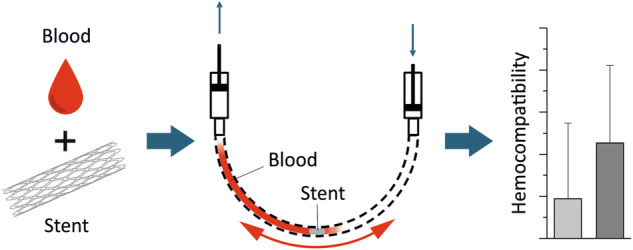

## Introduction

The in vitro evaluation of blood compatibility is an essential part of the development process of cardiovascular implants. Direct contact of biomaterials with blood leads to activation of platelets, the coagulation cascade and the complement system, as well as to haemolysis [1, 2]. According to ISO standard 10993-4, flow-dependent processes in particular play an important role [1, 3, 4], which is why the material or product to be tested should be brought into contact with blood under controlled and physiologically relevant flow conditions [5].

For this purpose, the blood must be moved over the test object in a very controlled manner in a sample chamber (i.e., tube) appropriately dimensioned for the test object. Furthermore, to prevent shear rate-dependent processes from superimposing or falsifying the result, the shear forces which occur during the pumping process must be low. Due to their high shear stress, conventional syringe or roller pumps are therefore unsuitable for such procedures from the outset. Alternative approaches such as ball valve pumps or gravity-driven systems (e.g., Chandler Loop) allow low-shear propulsion but are limited in the achievable flow rate in thin tubes [3].

We present here an in vitro test system for coronary stents and other cardiovascular medical devices that overcomes the disadvantages of previous test systems and thus can precisely and robustly map the influence of device geometry on flow-dependent blood reactions.

**Fig. 1:**
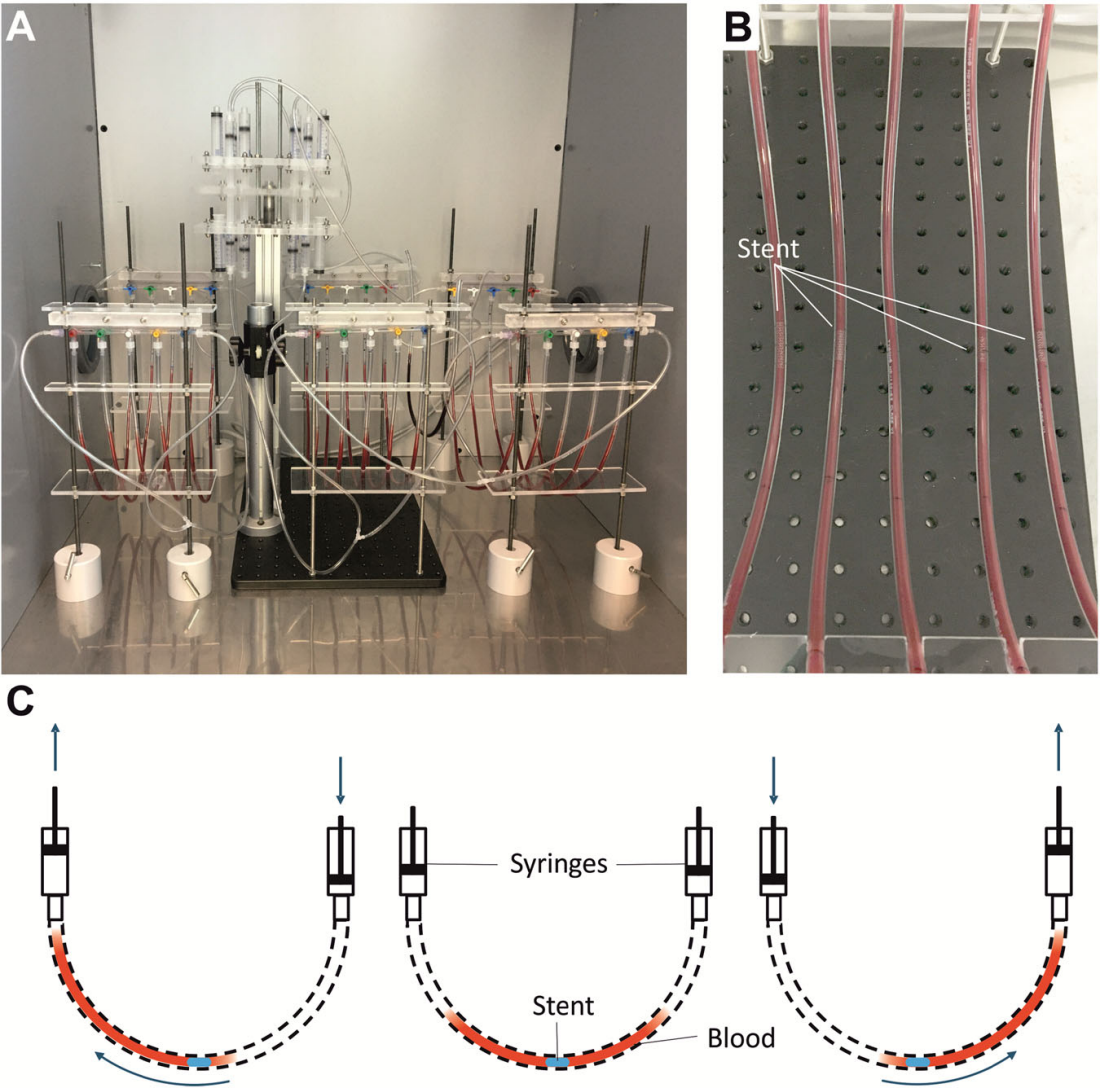
**A** *AirDrive* technology with 15 sample chambers. **B** Sample chambers with stent. **C** Schematic representation of the *AirDrive* technology

Our novel *AirDrive* technology (Fig. 1) involves an electric cylinder with opposing syringes mounted on the shaft of the cylinder. In operation, the (air-filled) syringes are compressed on one side while the opposite side is decompressed. The stents to be tested are fixed in flexible polyvinyl chloride (PVC) tubes similar to the implantation in the blood vessel (or other in vitro procedures), which are then filled with fresh human blood and connected to the pneumatic system. By alternately applying the tube openings to compressed air, a pulsating blood flow is created in the test chambers. Owing to the innovative drive mechanism, the blood flow over the test object can be adjusted very precisely by the compression speed and the amount of compressed air to create various flow velocities. Unlike other approaches, the test objects are constantly immersed in blood and at no time exposed to air. The cycle is repeated until the desired exposure time is reached, and then blood parameters of interest are quantified.

The current set-up allows the simultaneous measurement of up to 15 different sample chambers, five of them always connected in parallel to one pair of syringes. As a consequence, our test system offers the possibility to examine the test objects at up to three different velocities in parallel. Easy access to the sample chambers, which are not moved during the test itself, simplifies sampling for testing with different measuring times.

By quantifying the shear-dependent blood reactions to polished and unpolished versions of the same stent type in terms of haemolysis, platelet count and TAT generation, we demonstrate the robustness, precision and sensitivity of our approach.

## Materials and methods

### Blood collection

Fresh human whole blood was obtained in cooperation with the Medical Care Centre for Blood and Cancer Diseases in Potsdam, Germany, as part of a phlebotomy therapy of haemochromatosis patients. The blood was taken by means of a 15G venepuncture cannula according to Strauss into a blood bag specially made for this purpose. This transfusion bag, developed in cooperation with Meise GmbH, Schalksmühle, Germany, is a custom design with two separate bags.

Epithelial cells can detach from the patient’s skin during the puncture and thus falsify the subsequent blood tests. The first millilitres of blood were collected in a small extra bag at the time of collection to separate the epithelial cells. The blood used in the experiments was then directed into the second bag. For each donation, 500 mL of blood was collected and anticoagulated with a clinical dose of heparin (1 IU mL^−1^). During phlebotomy, the transfusion bag was continuously moved on a shaker to ensure uniform mixing of the blood with heparin. The experiments were performed within the first four hours after blood collection to ensure sufficient function of the blood components.

Blood donors were both male and female and between 45 and 65 years of age. For data protection reasons, no further data on the donors’ medical history or medication were collected.

### In vitro incubation

Hemocompatibility testing of CoCr-based polished and unpolished coronary stents with the same geometrical design (Cortronik GmbH, Rostock, Germany) was performed in a temperature-controlled incubator at a continuous 37 °C. DEHP-free PVC tubing (Tygon®-ND 100-65 (DEHPfree), 3.2 × 6.4 mm, ProLiquid, Germany) with an inner diameter of 3.2 mm and a length of 75 cm was used as a vessel imitation. The stents under investigation were placed in advance in the centre of the test tubes using balloon catheters (Pantera LEO 3.5/30 and Pantera PRO 3.0/30, Biotronik, Bülach, Switzerland). The tubes were subsequently filled with 3 mL of fresh blood. According to the manufacturer, typical values for the surface roughness of the inner side of polished and unpolished stents were approx. 80 and 350 nm, respectively.

The flow parameters were defined by the settings of the electric cylinder and the diameter of the used syringe type. The electric cylinder was controlled using the Festo configuration tool with the following parameters fixed for all experiments: movement distance = 40 mm, velocity = 50 mm s^−1^, acceleration = 12 m s^−2^, additional load = 1750 kg and deceleration = 5 ms. Comparative measurements between unpolished and polished stents were performed over various periods of time, as indicated. Depending on the type of syringe used (either 5, 10 or 15 mL volume), the above movement parameters of the electric cylinder resulted in an average blood flow velocity of 180, 340 or 560 mm s^−1^ (Fig. 3). To determine background activation, one tube without a stent was included as a negative control (NC) for each experiment. All blood parameters were determined as baseline values before the respective experiments (time *t* = 0) and after the termination of the experiment.

#### Roller pump closed-loop system

Prior to the arrival of the blood samples to the lab, the flow velocities applied by the roller pump were measured using a blood analogous fluid mixture (see below) and a PVC tube (Raumedic noDOP®, 3.2 × 6.4 mm, Raumedic, Germany) with a length of 60 cm, which was placed inside the roller pump (Watson Marlow) (note that we used the same tube type in the *AirDrive* model for comparative study). After the calibration, a new tube with the same length was placed inside the pump, and it was filled with approximately 4.5 mL of fresh human blood at a flow rate of 1 mL min^−1^. When there was no air remaining inside the tube, both ends were joined using an externally placed tube connector. Then, the flow rate was slowly increased until it reached the desired flow rate and the pump was placed in a temperature-controlled incubator at a continuous 37 °C.

### Sample handling

After the end of the experiment, the blood was emptied from the tubes into 15 ml cell culture tubes and 1000 µL of each blood sample was transferred into LoBind reaction tubes together with 50 μL of 500 mM EDTA or citrate (mixing ratio 1:10). A sample was taken from the EDTA whole blood to determine the platelet count and then the EDTA blood was centrifuged at 4000 rcf (Biofuge pico, Heraeus) for 2 min. The plasma supernatant was aliquoted and stored at −20 °C until further analysis. Citrate was mixed with blood at a ratio of 1:10 and centrifuged at 2500 rcf for 20 min after careful inversion. Plasma fractions were transferred to new reaction tubes and shock frozen with liquid nitrogen and stored at −20 °C until analysis. To evaluate flow-induced effects, baseline values of all analysed blood parameters were determined before each experiment.

#### Analysis of haemolysis

Free haemoglobin in platelet-poor plasma (PPP) was used as an indicator of haemolysis and was determined spectrophotometrically (Nanodrop 2000, Thermo Scientific, Darmstadt, Germany) using the Harboe method [6]. Using a previously established standard linear equation, the plasma haemoglobin concentration *cHb*_*Plasma*_ [mg mL^−1^] was calculated from this. To be able to relate the plasmatic haemoglobin concentration to the haemoglobin concentration of whole blood, the total haemoglobin concentration cHb_total_ [mg mL^−1^] was determined. For this purpose, whole blood was mixed 1:100 with Red Blood Cell (RBC) lysis buffer, placed in a rotator for 24 h, and then measured photometrically. To correlate the haemolysis of blood from different patients, the plasmatic haemoglobin concentration was corrected for the percentage of blood plasma (1 - H_tc_). Percentage haemolysis was calculated using the following formula:

$$H=\frac{{c}_{{{Hb}}_{{Plasma}}}x(1-{H}_{{tc}})}{{c}_{{{Hb}}_{{total}}}}$$

Finally, the percent haemolysis was subtracted from the corresponding reference sample at time *t* = 0. The difference (ΔH) indicates the flow-induced haemolysis.

#### Manual platelet counting in Neubauer chamber

Platelet counting was performed with EDTA whole blood within 24 h after collection. Samples were stored until analysis at 4–8 °C. EDTA blood was diluted 1:20 with Thrombo Count reagent and incubated for 30 min until complete lysis of erythrocytes, and disaggregation and rounding of platelets. The sample was filled into a Neubauer chamber and allowed to sediment for 20 min under humid conditions prior to light microscope inspection at 40× magnification. In the inner counting cross of the Neubauer chamber, five of the group squares were counted according to a fixed scheme. Platelet counting was performed in duplicate in each case.

#### Determination of TAT complexes

The concentration of thrombin-antithrombin III complexes (TAT) was analysed as an indicator of clot activation. For quantification of TAT in plasma from citrated blood, we used the Enzygnost® TAT micro-immunoassay (Siemens Healthcare Diagnostics Products, Marburg, Germany). Samples were applied undiluted, and further processing of the samples and standard solution was performed according to the manufacturer’s instructions. Absorbance was measured at a wavelength of 492 nm using a spectrophotometer (EnVision Multimode Microplate Reader, PerkinElmer, Waltham, USA). The TAT concentrations in the samples were derived from the reference curve.

### Statistical analysis

Statistical differences between means were tested for normal distribution using the Shapiro–Wilk test (computer program R) and analysed using the two-tailed non-parametric Mann–Whitney *U*-test. We considered a *p*-value of less than 0.05 to be statistically significant.

The means of the groups were tested for differences using one-way analysis of variance with repeated measures (rmANOVA), followed by a Tukey post hoc test for comparison of all pairs. *P*-values of <0.05 were considered significant.

### Velocity measurement

An optical measuring technique was developed in order to precisely measure the flow velocity inside the tubes during a cycle. For this purpose, a high-speed microscope camera system (Keyence VW-9000 equipped with VH-Z00R lens) was set to visualise the centre section of the tube.

The tubes were filled with 3 mL of a blood analogous fluid, which was made using a mixture of 40% PEG-200 and 60% distilled water [7]. The resulting density of the fluid was measured at 1.052 ± 0.002 g mL^−1^, and the reported viscosity was of 3.99 mPas. In one of the tubes, particles were mixed in with the fluid. To create the particles, a plastic-covered pink aluminium foil was cut into small squares in the order of sub-mm.

The movement of these particles was recorded using the microscope set at a frame rate of 2000 fps and an exposure time of 166 µs. Multiple combinations of different electric cylinder velocities and syringe volumes were tested while keeping the following parameters fixed for all experiments: movement distance = 40 mm, acceleration = 12 m s^−2^, additional load = 1750 kg and deceleration = 5 ms. The Keyence Motion Analyzer program was used to track about 20 particles corresponding to different locations along the cross-section of the tube. The velocity graphs obtained from each particle were joined to reconstruct the trajectory of the flow velocity over one cycle.

## Results

### In vitro-evaluation of geometry- and shear-dependent effects

The aim of our research was to develop a test system that allows the precise and robust investigation of the influence of important parameters of an implant, such as the implant geometry, on flow-dependent blood reactions. In a first study, we investigated whether the *AirDrive* test system is suitable to resolve geometry- and shear-dependent effects of stents of the same design but different surface finishes (polished vs. unpolished).

For that, polished and unpolished stents were placed into the test chambers and exposed to alternating blood flow with a flow velocity of 340 mm s^−1^. In addition, they were exposed to blood under static conditions. Empty tubes (i.e., without test objects) were used as a negative control (NC) for both static and dynamic conditions. After a test period of two hours, haemolysis, platelet activation and coagulation were determined and analysed. We conducted eight independent experiments with blood from four different donors. In order to be able to draw conclusions on the system-induced effects, static blood samples were analysed, too.

**Fig. 2:**
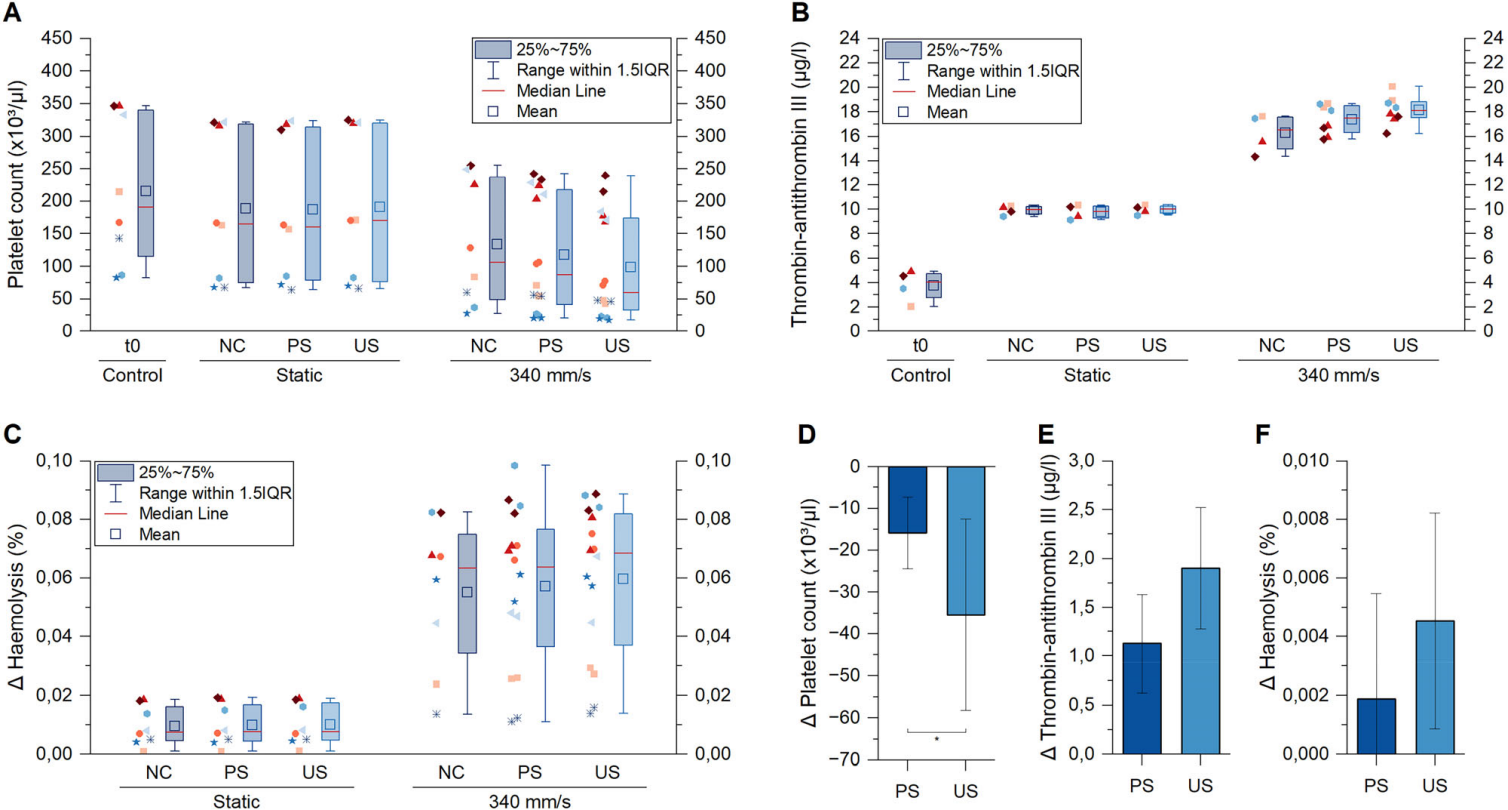
Effects of polished (PS) and unpolished (US) stents on **A** platelet activation **B** TAT concentration and **C** haemolysis after in vitro incubation at a mean flow velocity of 340 mm s^−1^ and an exposure time of 120 min. Empty sample chambers (NC) and static controls serve as reference values. Shown are the results from eight independent experiments with four different blood samples (mean haemolysis of the t_0_ control was 0.02% ± 0.009%). **D**–**F** shows the results from the dynamic sample chambers reduced by the negative control for better comparability. *Significant difference with *p* < 0.05

While the two stent types induced clearly different platelet activation and TAT concentration in the dynamic sample chambers (118,000 μL^−1^ vs 98,000 μL^−1^ and 17.4 μg L^−1^ vs 18.1 μg L^−1^ in polished and unpolished stents, respectively, Fig. 2A, B), no effect of the surface finish was observed in the non-moving controls (187,000 μL^−1^ vs 191,000 μL^−1^ and 9.8 μg L^−1^ vs 10 μg L^−1^, respectively). In ∆ haemolysis, the effects were less pronounced (at least at a flow velocity of 340 mm s^−1^), but a small difference between polished and unpolished stents was also visible here (i.e., 0.057% vs 0.06%, respectively Fig. 2C). Due to the heterogeneity of the different donor blood samples (indicated by colour and shape of data points in the figure), the data are subject to a high degree of scattering (note mean haemolysis of the t_0_ controls of the different blood samples was 0.02% ± 0.009%). Nevertheless, the results show that shear-dependent blood reactions of even single stents can be mapped with the *AirDrive* system.

Noteworthy is that moving the blood through the tubes induced considerable background activation of all tested parameters, as the dynamic negative control samples showed significantly lower platelet counts and higher haemolysis and TAT concentration than the static negative control (134,000 μl^−1^ vs 188,000 μl^−1^, 0.063% vs 0.007% and 16.3 μg L^−1^ vs 9.9 μg L^−1^, respectively, Fig. 2A–C). Therefore, for better comparability of the results, the negative control values associated with the respective experiments were subtracted (Fig. 2D–F). For platelet activation, a significant difference (*p* < 0.05) was detected between the polished and unpolished stents. This trend was also seen in the analysis of TAT concentrations and to a minor degree in the haemolysis, even though the latter two did not show statistical significance.

### Operation at different flow velocities

The flow velocity in the *Airflow* system can be easily set by adjusting the electric cylinder velocity or using syringes of different piston diameters. The latter also offers the possibility to apply several flow rates with the same electric cylinder set-up in parallel.

**Fig. 3:**
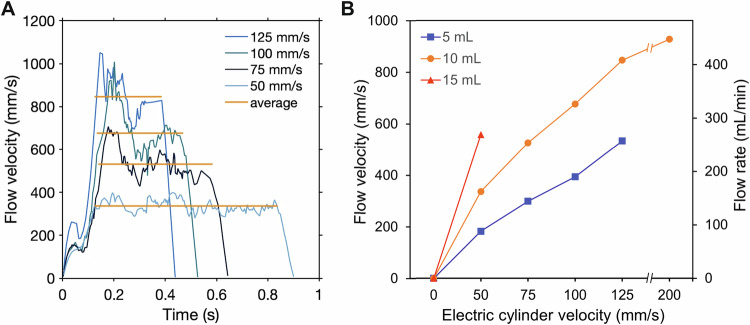
**A** Flow velocity and average velocity plotted over one cycle. The shown trajectories correspond to various electric cylinder velocities, 10 mL syringes and a movement distance of 40 mm. The trajectories were reconstructed from the tracking of various particles seeded in a blood analogous fluid. **B** Correlation between the set speed of the electric cylinder to flow velocity and rate for various syringe volumes

The flow velocity over a cycle was measured for different syringe volumes and electric cylinder velocities. For all parameter combinations, the course of the flow velocity over time shows an almost rectangular shape for each cycle (see Fig. 3A). The flow velocity initially exhibits a fast increase, reaching a peak between 0.1 s and 0.2 s after the start of a cycle. After this peak, it decreases slightly, staying within a certain range for most of the remaining cycle duration. At the end, the flow velocity decreases rapidly until it reaches a value near zero. As for the majority of the cycle, the velocity stays within a certain range, and the average during this section of the cycle was taken. Figure 3B shows the average flow velocities and flow rates for different parameter combinations. Depending on the parameters used, flow velocities up to 930 mm s^−1^, which corresponds to flow rates of up to 450 ml min^−1^ were possible. Note that for very high electric cylinder velocities (i.e., above 125 mm s^−1^), we observed a smaller flow velocity in the sample chamber than expected, possibly due to frictional forces occurring during the movement of the syringe piston, which increase excessively and counteract the movement of the piston.

The Reynolds number was calculated using the measured average flow velocities, and it was found to stay within a range of 155–930, indicating a laminar flow regime, as it stays below the critical Reynolds number for flow in circular tubes [8].

In order to demonstrate the operation of the *AirDrive* system at different flow velocities in the same experimental run (i.e., using the same donor blood sample), we equipped the electric cylinder with syringes of three different piston diameters and connected them to five tubes each. We placed polished and unpolished stents into the tubes and filled them with blood from the same donor. In addition, blood-filled tubes without samples under static or dynamic conditions served as a reference or represented the background activity of the dynamic model, respectively. We tested at flow velocities of 180 mm s^−1^, 340 mm s^−1^ and 560 mm s^−1^ for 120 min and analysed haemolysis and platelet count following blood exposure.

**Fig. 4:**
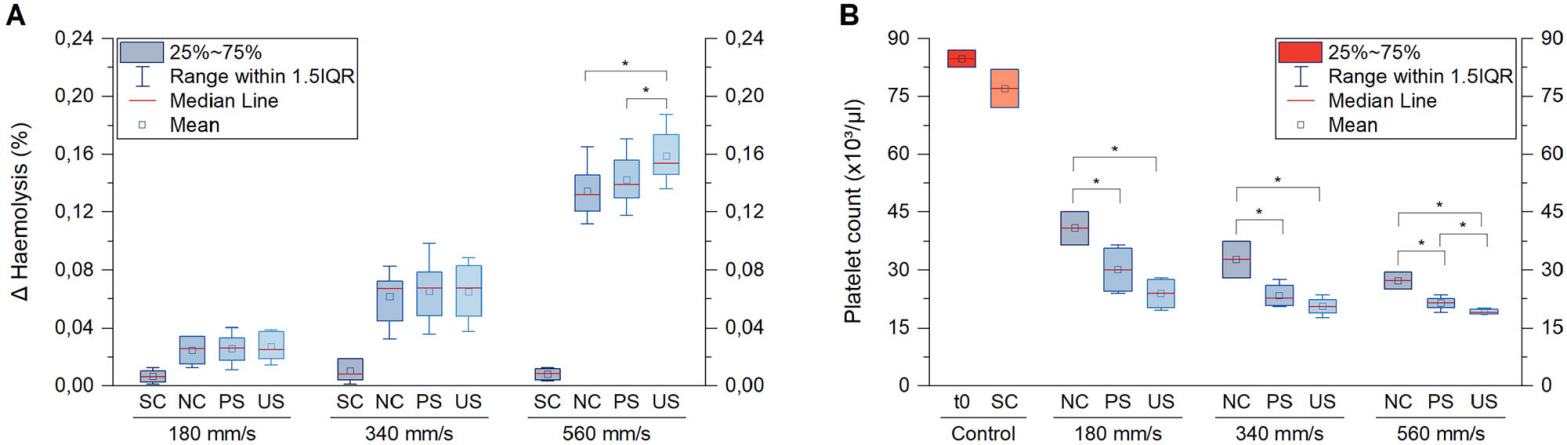
Operation of the *AirDrive* system at different flow velocities. Shear-induced **A** haemolysis and **B** platelet activation in relation to flow velocity. Polished (PS) and unpolished (US) stents were tested at different flow velocities for an exposure time of 120 min. Static controls (SC) and empty sample chambers (NC) served as reference values. Data from two independent experiments with two different blood donations

Our data show that the differences in blood response for the two types of stents become apparent at different velocities, depending on the blood parameter studied. For haemolysis, the smallest two velocities tested did not lead to a different blood reaction between polished and unpolished stents and differences became prominent only for the highest tested flow velocity (Fig. 4A). In contrast, differences in platelet counts were evident already at the smallest flow velocity tested (180 mm s^−1^), with the relative difference between polished and unpolished stents becoming more prominent with higher flow velocities (note that the platelet count in the donor blood was 85,000 µL^−1^ blood, which is far below the norm).

### Characterisation of the background activity and course measurements of blood parameters

In order to detect minimal differences in the response behaviour of the stents, it is important to know and take into account the background activation of the procedure for different test conditions.

**Fig. 5:**
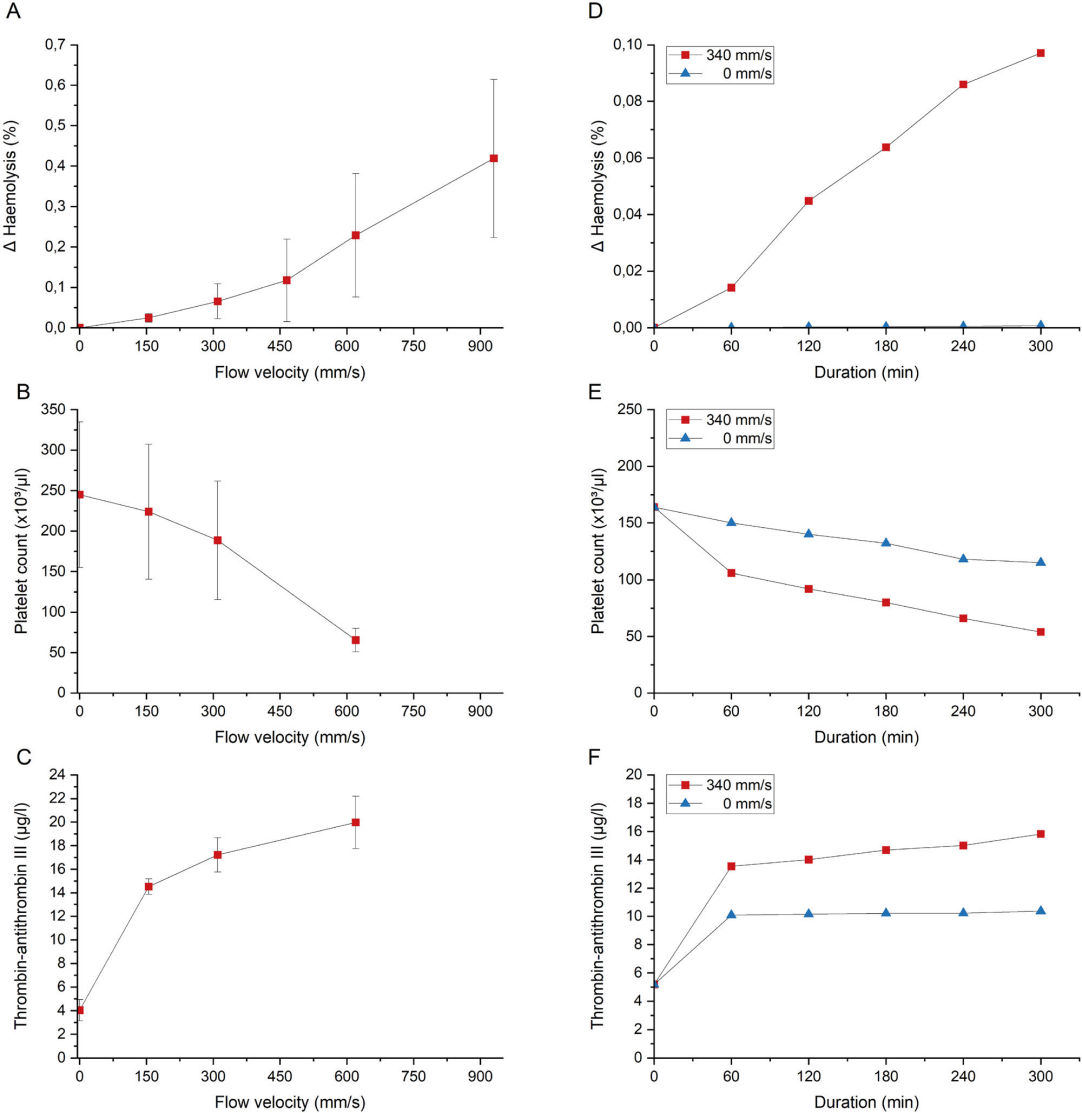
Characterisation of the background activity of the system in endpoint and course measurements. Sample chambers filled with blood were analysed either in an endpoint measurement at different flow rates or as a course measurement at a fixed flow rate (i.e., 340 mm s^−1^). For this purpose, haemolysis (**A**), platelet count (**B**) and concentration of TAT complexes (**C**) were determined. The mean values ± SEM from four experiments with three different blood samples are shown. Haemolysis (**D**), platelet count (**E**) and TAT concentration (**F**) were determined each hour over a period of 300 min. The static controls (0 mm s^−1^) showed lower values than the dynamic samples (340 mm s^−1^) for all parameters. The values from the dynamic and static sample chambers for one test are shown for comparison

For that, we tested the background activation at different flow velocities. The tests were carried out at flow velocities of 180 mm s^−1^, 340 mm s^−1^ and 560 mm s^−1^ over a period of 120 min. Afterwards, haemolysis, platelet count and concentration of TAT complexes were determined (Fig. 5A–C).

As expected, an increase in shear-induced haemolysis was observed with increasing flow velocity. At 180 mm s^−1^, Δ haemolysis was 0.02% ± 0.01% and increased to 0.42% ± 0.2% at 930 mm s^−1^. The relationship between flow velocity and haemolysis was not linear, as high velocities had a disproportionately stronger effect on erythrocyte lysis. In addition, the scatter of the measured values between the individual tests with different blood samples increased with increasing flow velocity. For the test series with polished and unpolished stents, we therefore avoided very high velocities above 560 mm s^−1^.

We also observed a velocity-dependency of platelet activation. Compared to the baseline value (245,000 ± 90,000 µL^−1^), the platelet count was reduced by 9% at a flow velocity of 180 mm s^−1^, whereas higher flow velocities led to a stronger activation of the platelets, so that at 930 mm s^−1^ the number of platelets had decreased to almost a quarter of the baseline value (65,500 ± 14,400 µL^−1^).

Coagulation activation was analysed by determining the TAT concentration. The TAT values also increased significantly compared to the baseline value (4 ± 0.9 µg L^−1^). Here, we observed the strongest increase between the initial value (t_0_) and the concentration at 180 mm s^−1^ (14.5 ± 0.7 µg L^−1^). Later experiments showed that this effect was predominantly triggered by the material contact during the first flow movements of the blood in the tube. However, the increase between the other tested velocities was much flatter up to a value of 20 ± 2.2 µg L^−1^.

To gain a better understanding of the temporal evolution of the blood reaction in the course of the experiment and to demonstrate that with our system, not only endpoint but also course measurement data can be collected easily, we analysed the blood parameters as a function of exposure time (Fig. 4D–F). To this end, we filled the tubes with blood and set the flow velocity to 340 mm s^−1^ or used them as a static control (0 mm s^−1^). After 60, 120, 180, 240 or 300 min, we collected blood samples and analysed them for haemolysis, platelet count and concentration of TAT complexes. In the dynamic condition, an increase in haemolysis was observed, corresponding to the duration of the experiment. After 60 min, the Δ haemolysis was 0.014%, increased continuously and reached a value of 0.097% after 300 min. The haemolysis triggered by shear stress in the sample chamber of the dynamic condition was significantly higher than the haemolysis in the static control without blood flow. Here, the Δ haemolysis was 0.001%. However, the shear-induced haemolysis with values less than 0.1% was, in general, very low even for the dynamic conditions and can be classified as non-haemolytic according to ASTM criteria [9].

With increasing exposure time, a decrease in the platelet count was observed in the static as well as in the dynamic condition. In the first 60 min, the decrease was more pronounced in the moving blood sample. After that, the gradient for the dynamic and the static condition is approximately the same. The platelet count decreased after 300 min from 164,000 µL^−1^ blood to 54,000 µL^−1^ blood in the dynamic sample and 115,000 µL^−1^ blood in the static control.

We observed a very similar trend in the coagulation activation. Also, the strongest increase in TAT concentration was observed at the beginning of the experiment. Within the first hour, the TAT concentration in the dynamic condition increased by a factor of 2.6 from 5.2 µg L^−1^ to 13.5 µg L^−1^. In contrast, the TAT concentration increased only slightly in the further time course (i.e., from 13.5 µg L^−1^ to 15.8 µg L^−1^ between 60 and 300 min). Coagulation activation also occurred in the static control, albeit less pronounced (i.e., 10.4 µg L^−1^).

### Comparison with roller pump

**Fig. 6:**
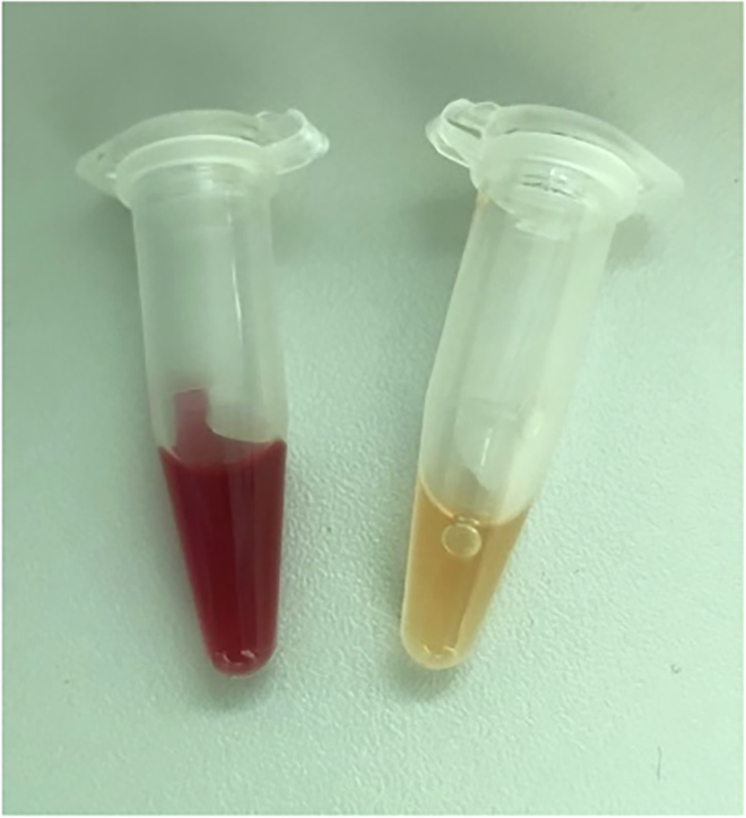
Plasma samples of blood obtained from tubes out of the roller pump closed-loop model (left) and the *AirDrive* system (right) after pumping blood at 330 ml min^−1^ (i.e., 680 mm s^−1^) through 3 mm diameter tubing for 180 min

Finally, we directly compared the background activation of our *AirDrive* system with a conventional roller pump closed-loop model for shear-induced haemolysis. For this purpose, we equipped both systems with tubes of the same type and manufacturer (i.e., Raumedic noDOP®, 3.2 × 6.4 mm, see “Materials and methods” section), filled them with the amount of blood required for the respective test system (i.e., 3 or 4.5 mL) and operated them without test objects at flow rates of 330 ml min^−1^ (i.e., 680 mm s^−1^). After a period of 180 min, we collected the plasma from the tubes and analysed the degree of haemolysis as described above. While Δ haemolysis was 1.47% for the roller pump closed-loop model, a significantly lower value of 0.24% was obtained with the *AirDriv*e system. This dramatic difference was clearly visible even to the naked eye (Fig. 6) as a strong contrast between the yellow liquid of the plasma from the *AirDrive* system and the red liquid resulting from the roller pump closed-loop model.

## Discussion

For dynamic hemocompatibility testing, animal models and in vitro test systems are used, the latter of which are much simpler to perform and also do not involve animal testing and associated (ethical) constraints. In contrast to animal models, in vitro models allow for precisely controlling flow conditions and other experimental settings, which increases robustness and reproducibility. They also offer the possibility of using human blood, which sometimes shows considerable differences in terms of blood coagulation and platelet function compared to animal blood [2, 10].

Today, a variety of in vitro test systems exist, with the modified Chandler loop and roller pump as the most common models [3, 11]. However, each model has shortcomings that limit its potential applications. With the aim of eliminating these shortcomings, we have developed a novel in vitro test model with an innovative drive.

Roller pumps generate high shear forces primarily due to the mechanical compression and movement of the tubing, which can lead to platelet activation and haemolysis (see Fig. 6). In contrast, the modified Chandler loop minimises blood damage by relying on a passive flow mechanism. However, the continual blood-air contact of the test object still leads to aggregation of platelets and denaturation of proteins [2, 12]. In addition, the air in the tube during rotation can cause the formation of foam and turbulence, resulting in uncontrolled flow conditions. Moreover, the Chandler loop is unsuitable for applications with high flow rates and small implants, as the entire tube content rotates at higher rotation speeds and the high surface-to-volume ratio that applies to small-diameter tubes [3]. Therefore, it cannot be used for measurements requiring tube inner diameters of about 3 mm (such as those used in our experiments) and flow rates ≥40 ml min^−1^ [3]. This does not even correspond to half of the right ventricular blood flow [13, 14] and is far below the value of ~250 ml min^−1^ (0.8 ml min^−1^ g^−1^ of myocardium) that describes coronary blood flow at rest (note this value can be increased even 9-fold during physical stress [15]).

With our *AirDrive* test system, high flow rates (up to 450 ml min^−1^) can be established even for small vessel lumens (see Fig. 2) without generating pump-related shear forces. In contrast to the Chandler loop, the test object in our test system is covered by blood during the entire test and, therefore, has no contact with air. This reduces the risk of system-induced activation processes on the coagulation cascade and increases the sensitivity and robustness of the test system. The blood volume of only three ml allows a high sample quantity and test run number with the same donor blood. This is an important aspect, especially for the comparability of the test results, as the blood of different donors shows a strong heterogeneity. With the *AirDrive* test system, we have developed a dynamic in vitro model that offers robust and controllable test conditions. Depending on the applied pressure, physiological flow profiles of high flow velocities and pulsed flow can be established.

**Fig. 7:**
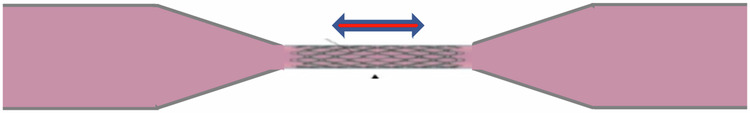
Adjustment of the tube geometry to reduce background effects

In our test system, the flow velocity is not constant over the entire test period, as in the Chandler loop or the roller pump. Rather, cyclical flow changes repeatedly both accelerate and decelerate the blood Fig. 7. As in real blood circulation, the maximum flow velocity is only reached for a short time. Physiological coronary blood flow is also phasic and is subject to very strong variations during the cardiac cycle. For example, the inflow into the left coronary artery varies from 10 ml min^−1^ at the end of systole to 150 ml min^−1^ at the beginning of diastole [13]. Although there is no reversal of the flow direction in vivo, we consider this deviation of our in vitro set-up from the physiological situation to be unproblematic, as long as the objects to be tested do not have a preferred direction (as is the case for the stents tested here).

Shear-induced blood reactions depend on both the flow conditions in the tube and the geometric properties of the implant. For blood compatibility testing of small coronary stents, this requires miniaturised test environments with small blood volumes. In this case, the ratio between the tube surface area and the blood volume becomes high, and the background activity induced by the surface of the blood-carrying components may mask the implant signal [16]. Therefore, to be able to measure an implant effect, the blood reaction caused by the test set-up itself must be as low as possible. Although we see some background activation of our test set-up, these effects apparently are small enough that the effects of a single stent can be distinguished from them (see Figs. 2 and 4).

In the direct comparison of polished and unpolished stents, the unpolished stents showed higher overall effects on the investigated parameters than the polished stents, with differences increasing with flow rate and an experimental duration (data not shown). Even if no statistical significance was shown for some cases or tested parameters, we observed a clear tendency, especially with regard to the coagulation and platelet parameters. Compared to the negative controls, there was a clear trend towards increased haemolysis and concentration of TAT complexes and decreased platelet counts for the stent-containing conditions. We observed a strong variability between the values from experiments with different whole blood donations, which reflects the different blood characteristics of the donors. However, this made the statistical evaluation difficult for test series carried out with different blood donations and only provided significant results with a high number of samples. The best comparison was made between values obtained within a series of tests carried out with the same whole blood. In future, working with pooled blood samples could help to reduce variability and thus improve statistical power. Future study donor variability could be reduced by pooling blood from different donors or narrowing the donor phenotype by the application of inclusion and exclusion criteria to the study (e.g., exclusion of donors on anti-coagulation therapy or similar).

Strategies to further reduce the background activation of our test set-up in future involve coating of the tube surface with anticoagulants like heparin [17–20] or other polymers, for example, TLP-coating [17, 21] and/or a changed tube geometry. Since background activation is a function of the blood-contacted surface area of the tubing and the flow velocity, it seems reasonable to us to expand the tube diameter upstream and downstream of the stent fixation (Fig. 6). This will decrease both the tubing surface area to blood volume ratio and the flow velocity in this area. As a result, the total shear stress and other mechanical forces acting on the blood components are reduced, and with it, erythrocyte damage and platelet activation.

The increasing mechanical shear at higher blood flow was particularly evident in haemolysis, which is very sensitive to mechanical forces [22, 23]. In addition to the level of haemolysis, the scatter of the values also increased with higher flow rates. However, the haemolysis induced by our system still can be considered as low, as the values were below 0.5% even after 120 min at a flow velocity of 930 mm s^−1^. So we can say that our system induces extremely low background activation in relation to haemolysis, similar to the results of the Haemobile model [24]. Test systems with peristaltic pumps, on the other hand, show significantly higher haemolysis values in haematology studies, triggered by the pump mechanism [3].

As coagulation involves adhesion and aggregation of platelets, we also analysed numbers of circulating platelets as a measure of blood compatibility. In our experiments, the number of free-flowing platelets decreased significantly in all sample chambers compared to the baseline level. However, only the difference with the dynamic sample chambers was statistically significant. These effects increased with the duration of the experiment and increasing flow velocity. The largest decrease was found in the first 60 min; after this time, the curve flattened out (Fig. 5E). Mueller et al. [25] also showed that the strongest effect was seen within the first hour in the modified Chandler Loop, as well as in the Haemobile model [24].

In Chandler loop experiments, the platelet count in the control was within the range of the baseline value [26, 27]. The greater impact of the model on platelet activation at the same flow rate compared to Chandler Loop experiments can be explained by the inner diameter of the sample chamber. Tubes with an inner diameter of 6.4 mm are often used in the Chandler Loop [28, 29]. However, smaller test chambers are necessary for testing coronary stents, which is why we used tubes with an inner diameter of 3.2 mm. A reduction in tube diameter, however, results in an increased shear stress [30] and with it in a higher platelet activation in the test system [24]. Engels et al. [31] studied the influence of the inner diameter of the sample chamber on the background activation induced by a dynamic test system. Experiments with the Haemobile model, which were also performed with a 3.2 mm tube, have shown a similar activation of platelets in the empty tube as in the results presented here [24].

The concentration of TAT complexes was determined as an indirect indicator of coagulation activation. In activated coagulation, thrombin binds to its physiological inhibitor, antithrombin III and forms a complex (TAT) with it. Overall, the TAT concentration increased significantly in all sample chambers compared to the baseline level. The strongest increase was measured in the first 60 min, and a weaker slope was registered in the further course of time. Even the static controls a doubling of the TAT concentration was observed after one hour. These values increased even more with a longer test duration. This result is consistent with the slight decrease in platelet counts in the static sample chamber. The reason for the onset of clotting in the static control may be, on the one hand, the condition of the tubes and, on the other hand, the heparin concentration used. Collected whole blood begins to clot immediately. This can be prevented by adding anticoagulants such as EDTA, citrate or heparin. To ensure that coagulation effects can still be measured, a very low heparin concentration (1 IU mL^−1^) was added to the whole blood for our experiments. In addition, effects caused by the non-physiological surface of the sample chambers are possible. In the dynamic sample chambers, the concentration of the TAT complex increased significantly more compared to the baseline and to the static control. The flow model itself, therefore, influences the coagulation activation. However, the highest values were measured for the sample chambers with unpolished stents.

Compared to other hemocompatibility systems, the *AirDrive* technology offers several advantages, which include the generation of robust and reproducible data, the low-shear propulsion of the blood over the test object, the very small sample volume, physiological flow profiles and high flow rates as well as the simple, cost-effective and adaptable handling to the sample geometry. Due to the variable speed setting and exposure time, our test system shows a high degree of freedom and is very well suited for the dynamic hemocompatibility testing of small vessel implants.

## Conclusion

Here, we report on a novel concept for blood flow generation in the context of hemocompatibility testing of small cardiovascular devices with only a few mm diameter, which can generate physiologically high flow rates (up to 450 mL min^−1^) without increased shear stress from the pump mechanism (*AirDrive* technology). In a proof-of-concept study, we were able to show that small geometric differences in stents (i.e., polished vs. non-polished surface) can be resolved by the system, as unpolished stents showed significantly reduced platelet numbers, as well as increased haemolysis and TAT activation, depending on flow velocity. The system allows working with only minimal blood volume (i.e., 3 mL per test) and the application of different flow rates in parallel. Together with the simple and cost-effective setup, this makes our *AirDrive* technology very well suited for dynamic hemocompatibility assessment of small vessel implants in the context of development and regulatory approval testing.
